# Global prevalence and pathogenesis of headache in COVID-19: A systematic review and meta-analysis

**DOI:** 10.12688/f1000research.27334.2

**Published:** 2021-03-10

**Authors:** Endang Mutiawati, Syahrul Syahrul, Marhami Fahriani, Jonny Karunia Fajar, Sukamto S. Mamada, Helnida Anggun Maliga, Nur Samsu, Muhammad Ilmawan, Yeni Purnamasari, Annisa Ayu Asmiragani, Ichsan Ichsan, Talha Bin Emran, Ali A. Rabaan, Sri Masyeni, Firzan Nainu, Harapan Harapan

**Affiliations:** 1 Department of Neurology, Universitas Syiah Kuala, Banda Aceh, Aceh, 23111, Indonesia; 2 Department of Neurology, Dr. Zainoel Abidin Hospital, Banda Aceh, Aceh, 23126, Indonesia; 3 Medical Research Unit, School of Medicine, Universitas Syiah Kuala, Banda Aceh, 23111, Indonesia; 4 Brawijaya Internal Medicine Research Center, Department of Internal Medicine, Faculty of Medicine, Universitas Brawijaya, Malang, East Java, 65145, Indonesia; 5 Faculty of Pharmacy, Hasanuddin University, Makassar, South Sulawesi, 90245, Indonesia; 6 Faculty of Medicine, Universitas Brawijaya, Malang, East Java, 65117, Indonesia; 7 Department of Microbiology, School of Medicine, Universitas Syiah Kuala, Banda Aceh, Aceh, 23111, Indonesia; 8 Department of Pharmacy, BGC Trust University Bangladesh, Chittagong, 4381, Bangladesh; 9 Molecular Diagnostic Laboratory, Johns Hopkins Aramco Healthcare, Dhahran, 31311, Saudi Arabia; 10 Department of Internal Medicine, Faculty of Medicine and Health Sciences, Universitas Warmadewa, Denpasar, Bali, 80235, Indonesia; 11 Department of Internal Medicine, Sanjiwani Hospital, Denpasar, Bali, 80235, Indonesia; 12 Tropical Disease Centre, School of Medicine, Universitas Syiah Kuala, Banda Aceh, Aceh, 23111, Indonesia

**Keywords:** COVID-19, SARS-CoV-2, headache, severity, predictor

## Abstract

**Background**: This study was conducted to determine the prevalence of headache in coronavirus disease 2019 (COVID-19) and to assess its association as a predictor for COVID-19. This study also aimed to discuss the possible pathogenesis of headache in COVID-19.

**Methods**: Available articles from PubMed, Scopus, and Web of Science were searched as of September 2 ^nd^, 2020. Data on characteristics of the study, headache and COVID-19 were extracted following the PRISMA guidelines. Biases were assessed using the Newcastle-Ottawa scale. The cumulative prevalence of headache was calculated for the general population (i.e. adults and children). The pooled odd ratio (OR) with 95% confidence intervals (95%CI) was calculated using the Z test to assess the association between headache and the presence of COVID-19 cases.

**Results**: We included 104,751 COVID-19 cases from 78 eligible studies to calculate the global prevalence of headache in COVID-19 and 17 studies were included to calculate the association of headache and COVID-19. The cumulative prevalence of headache in COVID-19 was 25.2% (26,464 out of 104,751 cases). Headache was found to be more prevalent, approximately by two-fold, in COVID-19 patients than in non-COVID-19 patients (other respiratory viral infections), OR: 1.73; 95% CI: 1.94, 2.5 with p=0.04.

**Conclusion**: Headache is common among COVID-19 patients and seems to be more common in COVID-19 patients compared to those with the non-COVID-19 viral infection. No definitive mechanisms on how headache  emerges in COVID-19 patients but several possible hypotheses have been proposed. However, extensive studies are warranted to elucidate the mechanisms.

**PROSPERO registration**: CRD42020210332 (28/09/2020)

## Introduction

The current coronavirus disease 2019 (COVID-19) pandemic has caused a global crisis for both the health and economic sectors. COVID-19 is caused by severe acute respiratory syndrome coronavirus 2 (SARS-CoV-2) which is a member of the *Coronavirinae* family and Betacoronavirus subfamily together with severe acute respiratory syndrome coronavirus (SARS-CoV), and Middle Eastern respiratory syndrome coronavirus (MERS-CoV) ^1^. The virus is primarily transmitted from person-to-person through droplets from symptomatic and pre-symptomatic patients, and is likely to also be transmitted by asymptomatic individuals ^2– 6^. Currently, no effective vaccines or pharmaceutical agents against SARS-CoV-2 are available but some progressions have been made to produce vaccines and drugs against the disease ^7– 10^.

Although up to 20.3% of hospitalized patients require admission to the intensive care unit (ICU) ^11^ with complications such as hypoxemia, acute respiratory distress syndrome (ARDS), arrhythmia, shock, acute cardiac injury, and acute kidney injury ^12– 14^, most SARS-CoV-2 infections are asymptomatic or have mild symptoms ^1, 15, 16^. The common clinical symptoms of COVID-19 include fever, dry cough, dyspnea, chest pain, fatigue and myalgia ^1, 12, 17, 18^. In some cases, other neurological manifestations such as headache, dizziness, seizure, taste and smell impairment were also reported ^12, 18– 21^. Headache is one of the symptoms that is also reported in various viral infections such as dengue and chikungunya that are common in the tropical regions ^22, 23^ and therefore may not be specific for COVID-19. In addition, the prevalence of headache in COVID-19 patients varies across studies ^19, 20, 24^. A study found that the prevalence of headache was 17.4% (94/540) in Hubei province, the epicenter of the outbreak, and 14.1% (111/788) among patients outside the epicenter ^21^. Another study in European countries found that the headache was reported in more than 40% of 417 COVID-19 patients ^19^. Furthermore, the association of headache with the presence of COVID-19 is unknown. This systematic review was undertaken to provide robust evidence on the prevalence of headache in COVID-19 patients globally and its association with COVID-19 cases. Information described in this study might help clinicians to decide whether headache could be used as one of the basic symptoms to be included in diagnosing SARS-CoV-2 infection, especially those in the front line with limited resources.

## Methods

### Registration and protocol

This systematic review was conducted as recommended by the Preferred Reporting Items for Systematic Reviews and Meta-analyses (PRISMA) guidelines ^25^. The protocol of this study was registered with PROSPERO, an international database of prospectively registered systematic reviews at the University of York, on 28 ^th^ September 2020 ( CRD42020210332).

### Eligibility criteria of studies 

Articles reporting headache as a symptom of COVID-19 cases were included. COVID-19 cases should be diagnosed with RT-PCR test using either nasopharyngeal and oropharyngeal swab, bronchoalveolar lavage or cerebrospinal fluid (CSF). All cross-sectional and cohort studies that included COVID-19 cases randomly selected from the population were considered eligible while case reports and case series including all editorials, reviews, and commentaries were excluded. Case-control studies with pre-allocated number of patients with headache and non-headache were excluded. Studies that were conducted in specific populations only such as in pregnancy, children, cancer patients and other groups were excluded. Only articles written in English were included in this study.

### Information sources and search strategy

To identify potential articles for analysis, systematic searches were conducted using three bibliographical databases ( PubMed, Scopus, and Web of Science as of September 2 ^nd^, 2020). The search criteria were as follows. Pubmed ([Title] "SARS-CoV-2" OR "COVID-19" OR "Wuhan coronavirus" OR "Wuhan virus" OR "novel coronavirus" OR "nCoV" OR "severe acute respiratory syndrome coronavirus 2" OR "coronavirus disease 2019 virus" OR "2019-nCoV" OR "2019 novel coronavirus" OR "severe acute respiratory syndrome coronavirus 2" OR "coronavirus" OR "coronaviruses" OR "SARS 2" OR "2019-nCoV acute respiratory disease" OR "novel coronavirus pneumonia"OR "COVID") AND ([All] “Headache”). Scopus ([Title] "SARS-CoV-2" OR "COVID-19" OR "Wuhan coronavirus" OR "Wuhan virus" OR "novel coronavirus" OR "nCoV" OR "severe acute respiratory syndrome coronavirus 2" OR "coronavirus disease 2019 virus" OR "2019-nCoV" OR "2019 novel coronavirus" OR "severe acute respiratory syndrome coronavirus 2" OR "coronavirus" OR "coronaviruses" OR "SARS 2" OR "2019-nCoV acute respiratory disease" OR "novel coronavirus pneumonia"OR "COVID") AND ([All] “Headache”). Web of Science ([Title] "SARS-CoV-2" OR "COVID-19" OR "Wuhan coronavirus" OR "Wuhan virus" OR "novel coronavirus" OR "nCoV" OR "severe acute respiratory syndrome coronavirus 2" OR "coronavirus disease 2019 virus" OR "2019-nCoV" OR "2019 novel coronavirus" OR "severe acute respiratory syndrome coronavirus 2" OR "coronavirus" OR "coronaviruses" OR "SARS 2" OR "2019-nCoV acute respiratory disease" OR "novel coronavirus pneumonia"OR "COVID") AND ([All] “Headache”). English as language limitation was imposed in the searches. Only peer-reviewed articles were included. Data were extracted both from the articles and the supplementary materials. Reference lists from the eligible articles were retrieved for further relevant studies.

### Study selection

All titles and abstracts of identified articles were imported into the EndNote X9 (Thompson Reuters, Philadelphia, PA, USA) and duplicate records between databases were removed. Retrieved articles were initially screened based on title and abstract to identify possible eligible studies. The full texts of potentially eligible articles were then reviewed. The screening and review processes were conducted by two authors (MF and JKF). After reviewing the full texts, the eligibility of each study was decided. Any discrepancies between the two authors were solved by consulting with another investigator (HH).

### Data extraction

The following data were extracted from eligible articles: study characteristics (author, title, journal, study site and study design), headache characteristics (number of patients with headache, type of headache, localization, and severity), COVID-19 characteristics (number of patients with COVID-19, severity, and outcome).

### Role of the funding source

This study received no external funding.

### Outcomes

The primary outcomes of this systematic review were: a) the prevalence of headache in COVID-19 cases; and b) the association between headache and COVID-19 cases compared to other viral infections.

### Data synthesis

The cumulative prevalence rate of headache was calculated for COVID-19 cases in the general population. The prevalence was calculated as the number of COVID-19 cases with headache divided by the total number of COVID-19 cases with and without headache, expressed as a percentage (%). Pooled odd ratios (OR) and 95% confidence intervals (95% CI) were calculated to assess the association of headache and COVID-19 compared to non-COVID-19 cases (other respiratory viral infections such as rhinovirus, influenza, parainfluenza and respiratory syncytial virus).

### Risk of bias assessment

To reduce sample selection bias, a critical assessment was specifically conducted in terms of setting of study and diagnosis of COVID-19. The quality of eligible studies was assessed using critical appraisals based on the Newcastle-Ottawa scale (NOS) ^26^. This scale evaluates 9 criteria of the study including the sample selection (4 items), group comparison (1 item), and the outcome (3 items). The scores range between 0 to 9 in which classified into three groups: low (≤ 4), moderate (between 5–6), and high quality study (≥ 7).

### Statistical analysis

The association between headache and the presence of COVID-19 was assessed by the calculation of a pooled OR and 95%CI using the Z test (p<0.05 was considered statistically significant). Prior to analysis, gathered data from studies were evaluated for heterogeneity and potential publication bias. Heterogeneity among studies was assessed using the Q test. Initial analysis found that the data had heterogeneity (p<0.10) and therefore a random effect model was employed. Egger’s test and a funnel plot were used to assess the reporting or publication bias (p<0.05 was considered having potential for publication bias). The data were analyzed using Review Manager version 5.3 ^27^. The cumulative pooled OR and 95%CI was presented in a forest plot.

## Results

### Study eligibility results

**Figure 1.**
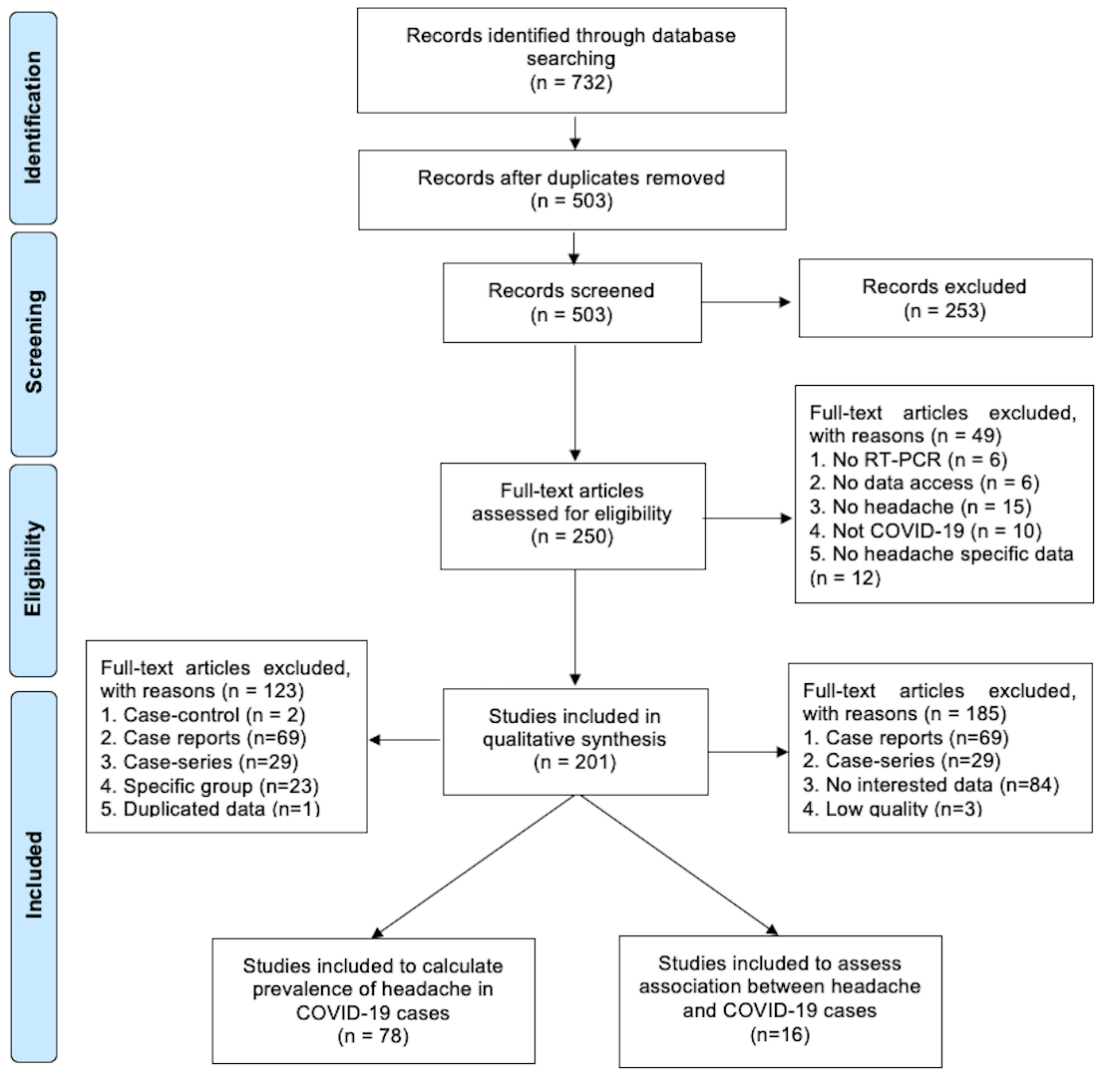
Flowchart of the result of literature search according to the preferred reporting items of systematic reviews and meta-analyses (PRISMA).

The literature searches yielded 732 articles, of which 229 were excluded as duplicates between databases. Following a screening process of the titles and abstracts of the remaining 503 articles, an additional 253 articles were excluded due to irrelevant studies leaving 250 articles ( Figure 1). The full texts of the remaining 250 articles were retrieved and screened for eligibility. This process excluded additional 49 articles that were not eligible as they did not fulfill the inclusion criteria. A full assessment was conducted on 201 articles. All included studies had high quality with NOS score ≥ 7.

To calculate the prevalence of headache in COVID-19, full-text assessment resulted in the exclusion of 123 articles for the following reasons: case control studies (n=2), case report studies (n=69), case series (n=29), duplicated dataset (n=1), and conducted in specific population only (n=23). The targeted population studies were conducted among healthcare workers ^28– 31^, diabetic patients ^32^, pediatrics ^33^, pregnant women ^34^, cancer patients ^35^, children and young adults ^36– 40^, patient undergone surgery ^41^, critical patients ^42^, women undergone delivery ^43^, patients with mild-moderate COVID-19 ^44– 46^, patients with gastrointestinal symptoms ^47^, patients with severe headache only ^48^, and patients with anosmia only ^49^. In total, 78 studies were included to calculate the prevalence of headache in COVID-19 and all studies were published in 2020. The studies were conducted in Brazil ^50^, China ^13, 51– 96^, Egypt ^97^, France ^98– 101^, Germany ^102^, India ^103, 104^, Italy ^105– 111^, Japan ^112, 113^, Jordan ^114^, Somalia ^115^, South Korea ^116, 117^, Spain ^118, 119^, Turkey ^120, 121^, and the US ^122– 126^. Two studies were cross-sectional ^119, 122^, five were prospective cohort studies ^65, 90, 98, 110, 121^ and the remaining 71 studies were retrospective studies.

To calculate the association between headache and COVID-19, the full-text assessment yielded 16 eligible studies. The rest of the references had been excluded for these reasons: (a) the studies were case reports or case-series (n=98); (b) the full-text did not include data of outcome of interest (n=84); and (c) low quality of study (n=3) ( Figure 1). The included studies were conducted in a wide ranges of regions: Australia ^127^, Belgium ^29^, Brazil ^50^, China ^70^, France ^99^, Hongkong ^128^, Israel ^129^, Italy ^130^, Germany ^131^, Netherlands ^31^, Turkey ^120^, and the US ^28, 105, 122, 132, 133^. Out of the studies, ten were case-control ^28, 29, 31, 99, 105, 120, 128, 130, 132^, four were cross-sectional ^50, 70, 122, 131, 133^, and two were prospective cohort studies ^127, 129^.

### The prevalence of headache in COVID-19 cases

Our systematic review included 78 studies consisting of 104,751 COVID-19 patients and headache was reported in 26,464 patients with a cumulative prevalence of 25.26%. The list of the studies and the prevalence of headache of each study is presented in Table 1. One study which included 51 patients described the specific location of headache: 1.96% (1/51) was a temporal headache, 35.29% (18/51) was a frontal headache, 23.52% (12/51) was a retro-orbital headache, and 39.21% (20/51) was a diffuse headache ^99^. Another study which involved 46 patients reported that 40 (86%) had tension-type pain and 6 (14%) had migraine-like headache ^107^. Data from 18 studies indicated that 72.17% (236/327) of headaches were reported in mild-moderate COVID-19 cases ^51, 53, 55– 58, 62, 63, 67, 76, 88, 92– 97, 117^. The prevalence of headache in severe COVID-19 cases from 15 studies was 27.83% (86/309) ^53, 55, 57, 58, 62, 63, 73, 76, 88, 92, 93, 95– 97, 117^.

**Table 1.** Characteristic of eligible studies and the prevalence of headache in each study.

No	Country	Study design	Total headache	Total population	Headache Percentage	Reference
1	Japan	Retrospective	7	57	12.3	112
2	Italy	Retrospective	10	213	4.7	105
3	China	Retrospective	4	72	5.6	51
4	USA	Cross sectional	39	59	66.1	122
5	China	Retrospective	6	83	7.2	52
6	Italy	Retrospective	11	70	15.7	106
7	China	Retrospective	17	262	6.5	53
8	USA	Retrospective	24,308	91,412	26.6	123
9	Egypt	Retrospective	18	66	27.3	97
10	India	Retrospective	34	522	6.5	103
11	China	Retrospective	2	14	14.3	54
12	China	Retrospective	5	50	10.0	55
13	India	Retrospective	3	21	14.3	104
14	China	Retrospective	3	36	8.3	56
15	China	Retrospective	2	20	10.0	57
16	China	Retrospective	19	189	10.1	58
17	China	Retrospective	3	37	8.1	59
18	South Korea	Retrospective	54	172	31.4	116
19	China	Retrospective	12	67	17.9	60
20	China	Retrospective	13	137	9.5	61
21	China	Retrospective	14	204	6.9	62
22	China	Retrospective	17	221	7.7	63
23	China	Retrospective	4	85	4.7	64
24	France	Prospective	99	197	50.2	98
25	France	Retrospective	51	70	72.9	99
26	China	Prospective	3	38	7.9	65
27	China	Retrospective	21	62	33.9	66
28	China	Retrospective	12	202	5.9	67
29	USA	Retrospective	72	251	28.7	124
30	China	Retrospective	9	85	10.6	68
31	China	Retrospective	75	788	9.5	69
32	Jordan	Retrospective	14	81	17.3	114
33	China	Retrospective	3	34	8.8	70
34	Japan	Retrospective	2	23	8.7	113
35	China	Retrospective	8	72	11.1	71
36	China	Retrospective	14	108	13.0	72
37	Italy	Retrospective	46	108	42.6	107
38	China	Retrospective	2	11	18.2	73
39	China	Retrospective	8	51	15.7	74
40	China	Retrospective	8	99	8.1	13
41	China	Retrospective	12	136	8.8	75
42	South Korea	Retrospective	140	694	20.2	117
43	China	Retrospective	5	48	10.4	76
44	China	Retrospective	4	28	14.3	77
45	China	Retrospective	14	53	26.4	78
46	China	Retrospective	11	125	8.8	79
47	China	Retrospective	67	651	10.3	80
48	China	Retrospective	98	1084	9.0	81
49	China	Retrospective	6	59	10.2	82
50	Spain	Retrospective	137	576	23.8	118
51	Spain	Cross sectional	104	576	18.1	119
52	France	Retrospective	82	139	59.0	100
53	China	Retrospective	21	270	7.8	83
54	China	Retrospective	80	655	12.2	84
55	China	Retrospective	2	33	6.1	85
56	Brazil	Retrospective	76	145	52.4	50
57	China	Retrospective	9	136	6.6	86
58	China	Retrospective	10	60	16.7	87
59	Somalia	Retrospective	10	60	16.7	115
60	Italy	Retrospective	2	10	20.0	108
61	Turkey	Retrospective	43	143	30.1	120
62	Germany	Retrospective	63	108	58.3	102
63	France	Retrospective	10	64	15.6	101
64	China	Retrospective	28	214	13.1	88
65	USA	Retrospective	129	208	62.0	125
66	Italy	Retrospective	30	72	41.7	109
67	Italy	Prospective	14	43	32.6	110
68	China	Retrospective	4	24	16.7	89
69	Turkey	Prospective	64	239	26.8	121
70	China	Prospective	3	8	37.5	90
71	China	Retrospective	35	187	18.7	91
72	China	Retrospective	5	93	5.4	92
73	China	Retrospective	3	29	10.3	93
74	China	Retrospective	1	108	0.9	94
75	China	Retrospective	20	663	3.0	95
76	USA	Retrospective	40	200	20.0	126
77	Italy	Retrospective	16	72	22.2	111
78	China	Retrospective	14	389	3.6	96
Total			26,464	104,751	25.2	

### Association of headache and COVID-19

**Figure 2.**
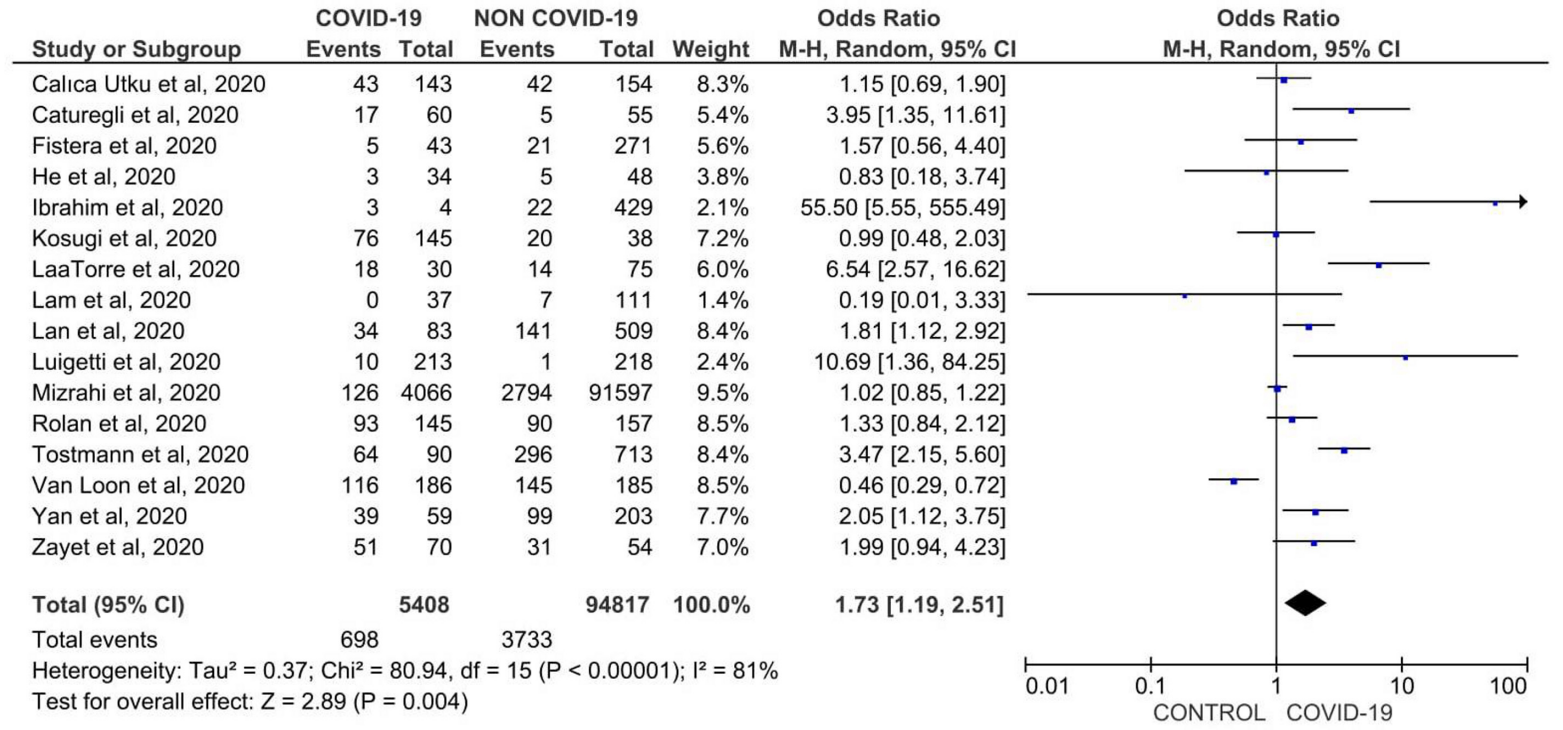
Forest plot of the correlation between headache and the prevalence of COVID-19.

A total of 16 studies, consisting of 5,407 COVID-19 cases in adults and 94,818 adults with non-COVID-19 infections (mostly COVID-19-like respiratory viral infections), were analyzed to determine the association between headache and COVID-19. Of these studies, an association between headache and the occurrence of COVID-19 was observed in 9 studies ^28, 29, 31, 105, 122, 127, 129, 130, 132^ while seven studies reported no association ^50, 70, 99, 120, 131, 133, 134^ ( Table 2). Our cumulative calculation revealed that headache was found to be 1.7-fold more prevalent in patients with COVID-19 compared to those with non-COVID-19 respiratory viral infections, OR: 1.73; 95% CI: 1.94, 2.51 with p=0.04. The correlation between headache and COVID-19 is presented in Figure 2.

**Table 2.** Prevalence of headache in COVID-19 and non-COVID-19.

Author, year	Study type	COVID-19		Non-COVID-19		COVID-19 severity	Control criteria	Reference
		Headache n (%)	Sample size	Headache n (%)	Sample size			
Çalıca Utku *et al.,* 2020	Case control	43 (0.30)	143	42 (0.27)	154	Mild-critical	Viral symptoms, negative PCR	120
Caturegli *et al.,* 2020	Case control	17 (0.28)	60	5 (0.09)	55	Not specified	Viral symptoms, negative PCR	132
Fistera *et al.,* 2020	Cross- sectional	5 (0.12)	43	21 (0.08)	271	Not specified	Viral symptoms, negative PCR	131
He *et al.,* 2020	Cross- sectional	3 (0.09)	34	5 (0.10)	48	Not specified	Viral symptoms, negative PCR	13
Ibrahim *et al.,* 2020	Cohort	3 (0.75)	4	22 (0.05)	429	Not specified	Viral symptoms, negative PCR	127
Kosugi *et al.,* 2020	Cross sectional	76 (0.52)	145	20 (0.53)	38	Not specified	Viral symptoms, negative PCR	50
La Torre *et al.,* 2020	Case control	18 (0.60)	30	14 (0.19)	75	Not specified	Viral symptoms, negative PCR	130
Lam *et al.,* 2020	Case control	0 (0.00)	37	7 (0.06)	111	Not specified	Viral symptoms, negative PCR	128
Lan *et al.,* 2020	Case control	34 (0.41)	83	141 (0.28)	509	Not specified	Viral symptoms, negative PCR	28
Luigetti *et al.,* 2020	Case control	10 (0.05)	213	1 (0.00)	218	Not specified	Viral symptoms, negative PCR	105
Mizrahi *et al.,* 2020	Cohort	126 (3.09)	4066	2794 (3.05)	91597	Not specified	Viral symptoms, negative PCR	129
Rolan *et al.,* 2020	Cross- sectional	93 (0.64)	145	90 (0.57)	157	Not specified	Viral symptoms, negative PCR	133
Tostmann *et al.,* 2020	Case control	64 (0.71)	90	296 (0.42)	713	Mild	COVID symptoms, negative PCR	31
Van Loon *et al.,* 2020	case control	145 (0.62)	185	116 (0.78)	186	Mild	Viral symptoms, negative PCR	29
Yan *et al.,* 2020	Cross- sectional	39 (0.66)	59	99 (0.49)	203	Not specified	Viral symptoms, negative PCR	122
Zayet *et al.,* 2020	Case control	51 (0.73)	70	31 (0.57)	54	Not specified	Confirmed influenza A/B	99

## Discussion

### Headache and COVID-19

As a non-specific symptom, headache might present not only in COVID-19 cases but also in other viral diseases, therefore, headache might not raise suspicion of SARS-CoV-2 infection ^22, 23^. However, a study described that headache is one of the main neurological symptoms of coronavirus infection including SARS-CoV-2 ^135^. The global prevalence of headache in our systematic review is more than 25% out of 104,751 COVID-19 cases. This result was almost double that of the previously reported prevalence from studies in China early in the pandemic that ranged from 6.5–13.1% ^53, 88^. This suggests that headache is prevalent in SARS-CoV-2 infection and therefore could potentially be used as one of the indicators to diagnose COVID-19 cases. In this analysis we did not analyze the prevalence of headache based on COVID-19 severity, the existence of COVID-19 co-morbidity (such as diabetes and hypertension) and based on demographic characteristics such as gender due to scarcity of the available data. Therefore, whenever enough data are available, such sub-analyses are critical to be conducted. 

Our study also highlights that headache was significantly more prevalent in COVID-19 patients, 2.2-fold, than suspected non-COVID-19 viral infection (other respiratory viral infections). A study found that only around 11% of MERS patients reported they suffered from headaches ^136^. A study in COVID-19 patients with pre-existing primary headache disorders revealed that the headache during COVID-19 had an unusual presentation with 42% (44/104) reporting a recent onset of headaches, 49% (51/104) had a change in headache pattern, and 39% (39/104) reported the worst headache they had ever had ^119^. These results suggest that new onset headache and changes of headache pattern should be carefully explored as this might be able to differentiate patients with COVID-19 from those without.

### Headache pathogenesis in COVID-19

We explored the available literature to broaden our knowledge of the pathogenesis of headache in COVID-19. In general, three main primary headaches are observed in COVID-19 patients i.e. migraine, cluster headache and tension-type headache ^137– 140^. No fixed mechanisms have been reported on how these headaches emerge in COVID-19 patients. However, it has been proposed that the activation of trigeminal nerve ending in the periphery followed by the sensitization of various sites in the brain is one of the main pathomechanism of headache in these patients ^137, 138^.

A headache attack is initiated by the release of several vasoactive neuropeptides such as glutamate, calcitonin gene-related peptide (CGRP), substance P and pituitary adenylate cyclase-activating polypeptide (PACAP), from nociceptive sensory fibers (especially nociceptive C-fibers and Aδ-fibers) innervating blood vessels located in the meninges and other cranial structures leading to vasodilation, degranulation of mast cells and plasma protein extravasation in those vascular structures ^141– 143^. The release of those peptides from the fibers could be due to either electrical, chemical or mechanical induction emerged from three branches of the trigeminal nerve i.e. ophthalmic, maxillary and mandibular branches ^143^. However, because of its wider area of innervation in the meninges and cranial blood vessels, ophthalmic branch seems to play more of a role in stimulating nociceptive processes in meningeal structures than the other two branches ^143, 144^.

Next, any physiological events, such as vasodilation, that have occurred in the meningeal and large cerebral blood vessels will become a stimulus which is sent to the trigeminal ganglion (TG) where other nociceptive information from other afferent trigeminal branches are also converging ^141^. Although cerebral and meningeal vasodilation is not the sole cause of headache ^145^, most studies have agreed on the critical role of blood vessel dilation in the emergence of headaches.

**Figure 3.**
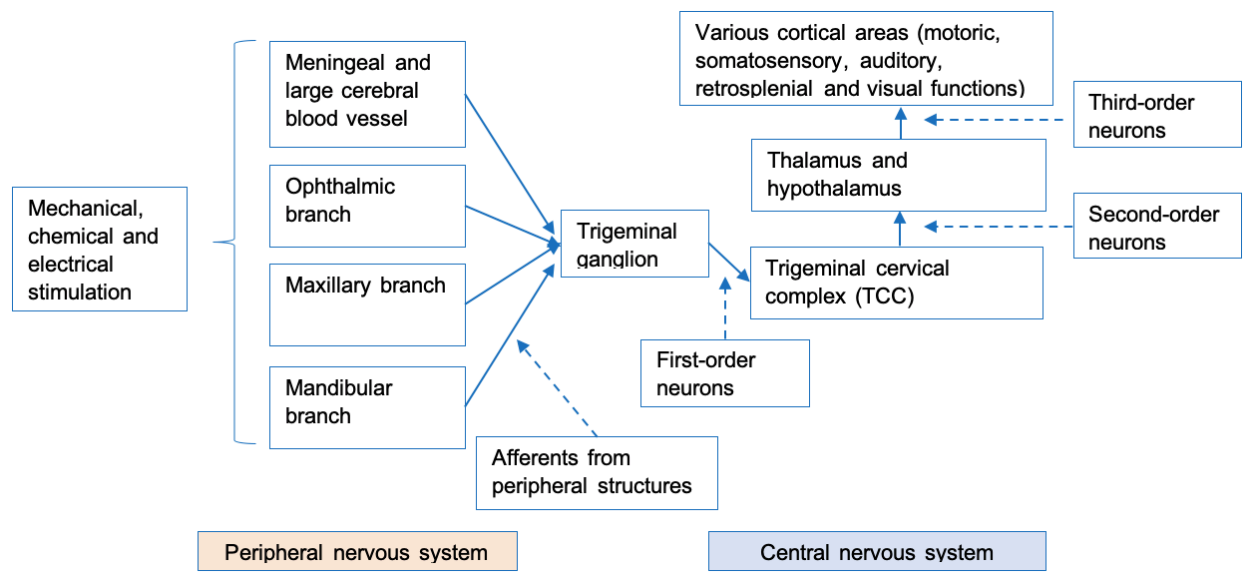
Proposed pathway of headache pathophysiology. Nociceptive information coming from peripheral networks is transmitted to trigeminal ganglion acting as central hub between peripheral and central nervous systems. Next, this information is sent to TCC located in the brainstem, transmitted to diencephalon structures and terminated in various areas in cortex. The transmission in this pathway is linked to the pivotal involvement of neurotransmitters (e.g. glutamate, GABA and serotonin) and nociceptive neuropeptides (e.g. CGRP, substance P and PACAP) released from nerve fibers synapses, particularly nociceptive C-fibers and Aδ-fibers. The receptors of these signaling molecules are identified in both peripheral blood vessel, trigeminal ganglion and central structures, such as in cerebrospinal fluid and TCC ^142, 147^.

From the TG, the stimulus is projected to an area in the brainstem called the trigeminocervical complex (TCC) via first-order neurons ^143^. These transmissions are then projected to the diencephalon structures, including the thalamus and hypothalamus, via the second-order neurons ^143, 146^. The third-order neurons are subsequently responsible for transmitting the information from diencephalic systems to various cortical areas associated with motoric, somatosensory, auditory, retrosplenial and visual functions ^141, 143^, leading to the manifestation of headache pain and other related symptoms ( Figure 3).

During these transmission processes, the release of neuropeptides, especially CGRP, is limited only in the meninges and in the central terminals of trigeminal afferents ^147^. When the transmission reaches TCC structures, CGRP and substance P may act to induce the release of glutamate and reduce gamma aminobutyric acid (GABA) production ^147, 148^. It has been proposed that during a headache attack, the level of glutamate in the TCC increases, while GABA release is decreased ^148^. This condition could result in the increase of nociceptive neurons excitability ^149^. Moreover, low level of serotonin in trigeminal nerve might also be involved in migraine pathophysiology as the release of this neurotransmitter has been linked to the inhibition of CGRP in trigeminal nerves ^150, 151^.

Several mechanisms have been postulated to explain how trigeminovascular system is activated in COVID-19. Firstly, direct invasion of the virus may activate the peripheral trigeminal system ^137^. This theory is hypothesized according to a study, confirming that trigeminal ganglia possess an angiotensinergic activity ^152^. Thus, the viral attack would hypothetically disturb the activity of the renin-angiotensin-aldosterone system (RAAS), which may increase the level of CGRP ^153^. Although the invasion of SARS-CoV-2 into the olfactory nerve ending seems to be the main route ^154– 156^, the action of the virus on the trigeminal nerve must not be overlooked, as suggested by Perlman *et al.* (1989). They demonstrated that the trigeminal nerve, in addition to the olfactory nerve, was a route used by neurotropic murine coronavirus to invade the central nervous system (CNS) ^157^. The hypothesis of trigeminal nerve attack by SARS-CoV-2 is also supported by the fact that olfactory mucosa is innervated by the trigeminal nerve ^158, 159^ suggesting the invasion of the olfactory mucosa by SARS-CoV-2 may also induce trigeminal nerve injury.

Following entry into the trigeminal nerve, SARS-CoV-2 may hijack the transneuronal transport system to direct the virus to enter the nucleus via a retrograde axonal transport mechanism. This transport occurs by the involvement of cytoskeletal motor proteins called dynein supported by cofactor dynactin that function to move substances, such as endosomes and vesicles, including hijacking viruses, on microtubule towards the cell body ^160^. Once the virus gains access to the nucleus in the cell body through the microtubule-organizing center (MTOC), viral replication is initiated ^161^. Finally, viral progenies may spread to other areas of the body, including the CNS, via anterograde axonal transport assisted by the kinesin motor protein family ^160^.

A study suggested a transneuronal transport system used by coronavirus after investigating the neuroinvasiveness of HCoV-OC43 in mice ^162^. Furthermore, the movement of SARS-CoV-2 via retrograde axonal transport is also hypothesized as it has been reported that the envelope protein of SARS-CoV could subvert dynein function either directly or indirectly ^163^. The role of dynein in the retrograde axonal movement of several viruses, such as herpesviruses, West Nile virus, rabies, and influenza virus, upon their penetration in the neuronal plasma membrane has also been reported ^164– 169^.

Secondly, SARS-CoV-2 may also invade the trigeminal nerve by indirect mechanisms. Cytokine storm and vasculopathy mechanisms are also proposed to explain the activation of trigeminal nerve upon SARS-CoV-2 infection ^137^. Cytokine storm has attracted substantial interest from researchers and clinicians as this unwanted condition is strongly suggested to be related to the increased mortality in SARS-CoV-2-infected patients ^170, 171^. It is hypothesized that the headache suffered by COVID-19 patients at the later stage of this infection is induced by the cytokine storm ^139, 172, 173^. This notion has been supported by the fact that proinflammatory cytokines, such as IL-1, IL-6 and TNF-α, have been linked to the activation of the trigeminovascular system, which is responsible for the emergence and development of headache through the modulation of CGRP ^174– 177^.

Moreover, the presence of angiotensin-converting enzyme 2 (ACE2) receptor on the endothelial cells makes the blood vessels vulnerable to invasion by SARS-CoV-2 ^178, 179^. It is known that ACE2 is associated with several protective mechanisms within the body, such as vasodilation ^180^ and antinociception ^181^. ACE2 also diminishes excessive free radical production which prevents oxidative stress ^182^. The utilization of this receptor by the virus may decrease its activities, leading to the disturbance of vascular function. The perivascular trigeminal nerve may in turn be affected resulting in the COVID-19-related headache ^137^. More studies are required to improve our understanding on the role of the ACE2 receptor in headache pathophysiology.

Another hypothesis by which SARS-CoV-2 could induce headache is offered by Abboud *et al.* (2020). They proposed that gas exchange disturbance in alveolar tissues triggered by the viruses would induce hypoxia, which in turn leads to ischemia ^170, 183^. Ischemia itself has been known to have a strong relation with headache incidents ^184^ that could be induced by exaggerating the production of free radicals.

In regards to the headache characteristics presented by COVID-19 patients compared to the headache induced by other viral infections, no apparent differences could be observed. Headache in COVID-19 could be worsened by physical or head movement, felt in either the entire head (holocranial) or unilaterally (hemicranial) and the pain is typically pressing or tightening ^139^. It is hypothesized that headaches occurring in COVID-19 patients might be the result of the same mechanisms as observed in influenza A and influenza B infections, which could be related to the activity of cytokines ^173, 185– 187^. A recent report on dengue-related headache suggested that the headache could be pulsating and either affect the entire brain, only frontal, or orbital area, which may resemble primary headaches reported in COVID-19 patients ^139, 172, 188^. Therefore, although we found that headache is more frequent in COVID-19 patients than those of non-COVID-19 patients, diagnosis of COVID-19 should not be based on the presence of a headache.

In conclusion, headache is a common symptom in COVID-19 cases. Some mechanisms have been proposed as to the mechanism for headaches in COVID-19 such as the activation of the trigeminovascular system by either direct action of the virus or indirect mechanisms induced by cytokine storm, vasculopathy, or ischemia induced by gas exchange disturbance in COVID-19 patients. Extensive efforts must be carried out to provide definitive answers about COVID-19-related headaches. Detailed investigations on the mechanisms by which SARS-CoV-2 attacks the CNS and thus generates headaches are important to improve our understanding on the pathophysiology of COVID-19, and therefore influences possible pharmacological intervention decisions.

## Data availability

### Underlying data

All data underlying the results are available as part of the article and no additional source data are required.

### Reporting guidelines

Figshare: PRISMA checklist for ‘Global prevalence and pathogenesis of headache in COVID-19: A systematic review and meta-analysis’, https://doi.org/10.6084/m9.figshare.13166783.v1 ^189^

Data are available under the terms of the Creative Commons Attribution 4.0 International license (CC-BY 4.0).
