# Supplementary material for: Global prevalence and pathogenesis of headache in COVID-19: A systematic review and meta-analysis
Source: F1000Res. 2021 Mar 10;9:1316. Originally published 2020 Nov 12. [Version 2] doi: 10.12688/f1000research.27334.2 (PMC8063523; doi:10.12688/f1000research.27334.2)
Supplement: Peer review history

# Peer review: Reviewer response for version 2

**DOI:** 10.5256/f1000research.55021.r81184

**Published:** 2021-04-14

**Authors:** Erni J. Nelwan

**Affiliations:** 1 Division of Tropical Medicine and Infectious Disease, Department of Internal Medicine, Medical Faculty, Cipto Mangunkusumo General Hospital, University of Indonesia, Jakarta, Indonesia; 2 Infectious Diseases and Immunology Research Cluster, Faculty of Medicine, Universitas Indonesia, Depok City, Indonesia

I confirm that I have read this submission and believe that I have an appropriate level of expertise to confirm that it is of an acceptable scientific standard, however, I have significant reservations, as outlined above.

Are the rationale for, and objectives of, the Systematic Review clearly stated?

Partly

Is the statistical analysis and its interpretation appropriate?

I cannot comment. A qualified statistician is required.

Are sufficient details of the methods and analysis provided to allow replication by others?

No

Are the conclusions drawn adequately supported by the results presented in the review?

Partly

Reviewer Expertise:

NA

I confirm that I have read this submission and believe that I have an appropriate level of expertise to confirm that it is of an acceptable scientific standard.

# Peer review: Reviewer response for version 2

**DOI:** 10.5256/f1000research.55021.r81183

**Published:** 2021-03-11

**Authors:** Morteza Arab-Zozani

**Affiliations:** 1 Social Determinants of Health Research Center, Birjand University of Medical Sciences, Birjand, Iran

Thank you for modifying your paper and clearly addressing my comments. In my opinion, the manuscript is acceptable.

 Cheers

Are the rationale for, and objectives of, the Systematic Review clearly stated?

Yes

Is the statistical analysis and its interpretation appropriate?

Yes

Are sufficient details of the methods and analysis provided to allow replication by others?

Yes

Are the conclusions drawn adequately supported by the results presented in the review?

Yes

Reviewer Expertise:

Health Policy and Management, an expert in systematic review and meta-analysis methodology.

I confirm that I have read this submission and believe that I have an appropriate level of expertise to confirm that it is of an acceptable scientific standard.

# Peer review: Reviewer response for version 1

**DOI:** 10.5256/f1000research.30205.r79512

**Published:** 2021-02-15

**Authors:** Morteza Arab-Zozani

**Affiliations:** 1 Social Determinants of Health Research Center, Birjand University of Medical Sciences, Birjand, Iran

Thank you for considering a great area of interest related to COVID-19. This study aimed to investigate the prevalence and pathogenesis of headache in COVID-19 patients, globally. The methodology is completely rigorous and the results are interesting. I have a few minor comments to the authors as follow:

- In the study selection and/or quality appraisal stages are the authors faced with discrepancies? Please state how resolved these potential discrepancies.
- Was it not possible to analyze the subgroups? For example, based on age groups or gender.
- Please add some details about the NOS checklist and also add the result of the quality appraisal to the manuscript.

Are the rationale for, and objectives of, the Systematic Review clearly stated?

Yes

Is the statistical analysis and its interpretation appropriate?

Yes

Are sufficient details of the methods and analysis provided to allow replication by others?

Yes

Are the conclusions drawn adequately supported by the results presented in the review?

Yes

Reviewer Expertise:

Health Policy and Management, an expert in systematic review and meta-analysis methodology.

I confirm that I have read this submission and believe that I have an appropriate level of expertise to confirm that it is of an acceptable scientific standard, however I have significant reservations, as outlined above.

# Response

**Published:** 2021-02-26

**Authors:** Endang Mutiawati

**Affiliations:** Universitas Syiah Kuala, Indonesia

Dear Dr Morteza Arab-Zozani, 

 Thank you for your comments.

 1. We have provide the approach how to solve in case there was discrepancies between two authors during evaluation of the studies. 

 2. We were unable to do sub-group analysis based on age and gender due to available of data.  We have added this as one of our limitation: We have added this in our manuscript: "In this analysis we did not analyze the prevalence of headache based on COVID-19 severity, the existence of COVID-19 co-morbidity (such as diabetes and hypertension) and based on demographic characteristics such as gender due to scarcity of the available data. Therefore, whenever enough data are available, such sub-analyses are critical to be conducted."

 3. We have provide more detail information of Newcastle-Ottawa scale (NOS) - What items that were assessed and how to score as well how the studies classified. We also have added the NOS scores briefly to all studies included in the study. 

 Thank you

# Peer review: Reviewer response for version 1

**DOI:** 10.5256/f1000research.30205.r74699

**Published:** 2020-12-04

**Authors:** Erni J. Nelwan

**Affiliations:** 1 Division of Tropical Medicine and Infectious Disease, Department of Internal Medicine, Medical Faculty, Cipto Mangunkusumo General Hospital, University of Indonesia, Jakarta, Indonesia; 2 Infectious Diseases and Immunology Research Cluster, Faculty of Medicine, Universitas Indonesia, Depok City, Indonesia

Authors seem not considered potential important bias while choosing headaches to be analysed. While among patients with fever (mainly), headache is in the same pathophysiology response due to prostaglandin release. Not to include other symptoms, so it is not merely a headache as a symptom.

 How about the existence of co-morbidity such as Hypertension (uncontrolled hypertension)? It is not clear how to justify data with such condition concomitantly.

 In the results section, what does this statement mean?

 "Headache was found to be more prevalent, approximately by two-fold, in COVID-19 patients than in non-COVID-19 patients with symptoms of other respiratory viral infections, OR: 1.73; 95% CI: 1.94, 2.5 with p=0.04."

 What kind of non-covid-19 patients with symptoms of other respiratory viral infections? This is a confusing statement.

 As a clinician taking care of Covid-19 patients, the importance of this article is low.

Are the rationale for, and objectives of, the Systematic Review clearly stated?

Partly

Is the statistical analysis and its interpretation appropriate?

I cannot comment. A qualified statistician is required.

Are sufficient details of the methods and analysis provided to allow replication by others?

No

Are the conclusions drawn adequately supported by the results presented in the review?

Partly

Reviewer Expertise:

infectious disease

I confirm that I have read this submission and believe that I have an appropriate level of expertise to confirm that it is of an acceptable scientific standard, however I have significant reservations, as outlined above.

# Response

**Published:** 2021-02-26

**Authors:** Endang Mutiawati

**Affiliations:** Universitas Syiah Kuala, Indonesia

Dear Dr Erni J. Nelwan

 We agreed that our study have limitation since we did not able to analysed the data based on existence of COVID-19 co-morbidity. This is mainly because the available of data. The data was scarce. 

 We have added this in our manuscript: "In this analysis we did not analyze the prevalence of headache based on COVID-19 severity, the existence of COVID-19 co-morbidity (such as diabetes and hypertension) and based on demographic characteristics such as gender due to scarcity of the available data. Therefore, whenever enough data are available, such sub-analyses are critical to be conducted."

 We have provided a clear explanation about the non-COVID-19 in Method section as requestioned. Not-COVID-19 in our study refers to other respiratory viral infections such as rhinovirus, influenza, parainfluenza and respiratory syncytial virus. We added: "non-COVID-19 cases (other respiratory viral infections such as rhinovirus, influenza, parainfluenza and respiratory syncytial virus)" in text. 

 We also also agreed that the pathogenesis of headache is complex including the production of prostaglandin while fever. Therefore in our study we compared the occurrence of headache in COVID-19 and other non-COVID-19 respiratory infection as control. This to reduce the effect of fever since in both COVID-19 and non-COVID-19 cases the fever are present. Due to this complexity we did not claim any diagnostic for direct clinical practice.
